# Multifocal Femto-PresbyLASIK in Pseudophakic Eyes

**DOI:** 10.3390/jcm10112282

**Published:** 2021-05-25

**Authors:** Bojan Pajic, Horace Massa, Philipp B. Baenninger, Erika Eskina, Brigitte Pajic-Eggspuehler, Mirko Resan, Zeljka Cvejic

**Affiliations:** 1 Eye Clinic Orasis, Swiss Eye Research Foundation, 5734 Reinach, Switzerland; brigitte.pajic@orasis.ch; 2 Department of Physics, Faculty of Sciences, University of Novi Sad, Trg Dositeja Obradovica 4, 21000 Novi Sad, Serbia; zeljka.cvejic@df.uns.ac.rs; 3 Department of Clinical Neurosciences, Division of Ophthalmology, Geneva University Hospitals, 1205 Geneva, Switzerland; horace.massa@hcuge.ch; 4 Faculty of Medicine, University of Geneva, 1205 Geneva, Switzerland; 5 Faculty of Medicine of the Military Medical Academy, University of Defense, 11000 Belgrade, Serbia; resan.mirko@gmail.com; 6 Cantonal Hospital of Lucerne, Department of Ophthalmology, 6006 Lucerne, Switzerland; philipp.baenninger@luks.ch; 7 Ophthalmological Department of Academy of Postgraduate Education FSBF FRCC of the FMBA of Russia, 125310 Moscow, Russia; erika.eskina@sfe.ru; 8 Laser Surgery Clinic “SPHERE”, 117628 Moscow, Russia

**Keywords:** presbyLASIK, excimer laser, multifocality, pseudophakic

## Abstract

Background: Presbyopia treatment in pseudophakic patients with a monofocal IOL is challenging. This study investigates the refractive results of femto-PresbyLASIK and analyzes presbyopia treatment in pseudophakic eyes. Methods: 14 patients with 28 pseudophakic eyes were treated with femto-PresbyLASIK. The dominant eye was targeted at a distance and the non-dominant eye at −0.5 D. The presbyopic algorithm creates a steepness in the cornea center by using an excimer laser that leads to corneal multifocality. Results: 6 months after surgery a refraction of −0.11 ± 0.13 D (*p* = 0.001), an uncorrected distance visual acuity of 0.05 ± 1.0 logMAR (*p* < 0.001) and an uncorrected near visual acuity of 0.15 ± 0.89 logMAR (*p* = 0.001) were achieved in the dominant eye. For the non-dominant eye, the refraction was −0.28 ± 0.22 D (*p* = 0.002), the uncorrected distance of visual acuity was 0.1 ± 1.49 logMAR, and the uncorrected near visual acuity was 0.11 ± 0.80 logMAR (*p* < 0.001). Spherical aberrations (Z400) were reduced by 0.21–0.3 µm in 32% of eyes, and by 0.31–0.4 µm in 26% of eyes. Conclusion: By steepening the central cornea while maintaining spherical aberrations within acceptable limits, PresbyLASIK created a corneal multifocality that safely improved near vision in both eyes. Thus, femto-PresbyLASIK can be used to treat presbyopia in pseudophakic eyes without performing intraocular surgery.

## 1. Introduction

While patients are increasingly aware of the possibility that they do not need to wear spectacles after cataract surgery, if they have a simultaneous multifocal intraocular lens (IOL) placed, few patients are benefiting from this advanced technology. Indeed, the vast majority of patients are choosing a monofocal IOL. Limitations to the wider use of multifocal IOL might be their high cost, the careful patient selection that is required for good outcomes, or the patients’ fear of side effects. Monofocal IOL placement after cataract surgery allows perfect vision, but only at one focal distance. This monovision can lead to patient dissatisfaction, and a desire to regain multifocality without corrective lenses. Unfortunately, solutions to restore multifocality remain scarce and poorly explored.

There are different ways to reach multifocality. The goal is to achieve the best possible visual outcome while maintaining a low level of optical disturbance. There are only 3 surgical options to treat presbyopia [1]. One option is to exchange the monofocal lens with a multifocal one, but this remains challenging, as treated eyes might be weakened by the first surgery. Thus, this does not have a high level of safety [2]. A second option would be the implantation of a multifocal add-on IOL [3], but this has several limitations. The power calculations of lenses are not as precise as with a laser. Deposits in the interface between the IOL in the bag and the add-on IOL might also disturb the vision. The rubbing of the add-on IOL against the iris tissues might induce ocular inflammation [4], or a pigment dispersion syndrome that risks elevated intraocular pressure and glaucoma. Lastly, this is an intraocular procedure that poses a certain amount of complication risk. The third option is to obtain multifocality at the corneal level. The concept of multifocal PresbyLASIK is an attractive correction method, because the surgical technique is based on the LASIK method. In contrast to a multifocal IOL implantation, minimal invasive surgery is necessary, because the eye does not need to be opened up. PresbyLASIK involves two steps. The first step is to correct ametropia for distance vision, and the second step is to make an addition for near vision. In multifocality, the central part of the cornea is most often adjusted for proximity, and the middle periphery is corrected for distance [5,6,7,8,9,10].

A conventional PresbyLASIK always represents a compromise between distance and near vision, since it creates unwanted aberrations, especially spherical aberrations in the central pupillary region. To minimize unwanted aberrations, today’s PresbyLASIK treatment algorithms are wavefront-guided. Compared to the well-established treatment of presbyopia with a multifocal IOL, PresbyLASIK is a newer surgical technique, but it has the advantage of being less invasive than implanting an IOL, because the eye does not need to be opened up. On the other hand, the use of PresbyLASIK is much more demanding. Patient selection and the interpretation of objective preoperative topographic and wavefront analyses are challenging. In particular, decisions based on the Zernike polynomial analysis of the cornea have a high influence on the surgical result.

It is not sufficient to use the general LASIK criteria for PresbyLASIK application. The lack of encouraging treatment results of PresbyLASIK to date is likely because the indication for surgery was on LASIK criteria, and corresponding refraction and other parameters from the wavefront analysis were not taken into account [11]. Moreover, even if wavefront analysis is done perfectly, it must be adapted to pupillary diameter in the mesopic condition [12].

The PresbyLASIK, in particular the Supracor algorithm, was already successfully used with presbyopic phakic eyes [11,13,14]. Therefore, in this retrospective study, we assessed refractive outcomes after PresbyLASIK in pseudophakic patients.

## 2. Materials and Methods

We included 14 patients with 28 pseudophakic eyes in this retrospective case series. The same surgeon performed all surgical procedures. The study was approved by the Ethics Committee in Novi Sad (34–08.18). This study was conducted in accordance with the Protocol, the Declaration of Helsinki, and all applicable regulatory requirements.

In the following Table 1, all significant preoperative PresbyLASIK data were listed of all 14 patients, such as mean age, UDVA, CDVA, UNVA, CNVA for the dominant and non-dominant eye, as well as the refraction preoperative levels for the dominant and non-dominant eyes. The mean period between cataract surgery and presbyopia was 8.93 ± 3.82 (range 6–16) months (Table 1).

Inclusion criteria were a dissatisfaction with myopic refraction obtained with a monofocal IOL with manifest refraction spherical equivalent (MRSE) between −0.5 and −4.0 diopters (D), astigmatism of 2.50 D or less, mean keratometry between 41.00 and 46.00 D, a central corneal thickness of 540 μm or more, a mesopic pupil diameter between 4 and 6 mm, and a kappa angle of less than 6°. Exclusion criteria were the presence of ocular surface disease, clinically significant corneal opacity, posterior segment ocular pathologies, and abnormal corneal topography. All pseudophakic patients received the same IOL (Nidek NS 60YG, Nidek CO. LTD., Gamagori, Japan) during the cataract surgery.

All patients had a complete ophthalmologic examination prior to surgery, including manifest refraction, cycloplegic refraction, slit lamp microscopy of the anterior ocular segment, dilated fundoscopy and intraocular pressure measurement. The preoperative examination also included corneal topography with the Orbscan II system (Technolas Perfect Vision GmbH, Munich, Germany) and Pentacam (Oculus optical devices, Wetzlar, Germany). Wavefront aberrometry measurements were performed preoperatively with the Zywave II aberrometer (Technolas Perfect Vision GmbH) with undilated pupils and pupillometry. Eye dominance was determined by means of a “hole test”. The measurement was carried out under scotopic conditions. The aberration analysis was carried out in a 6-mm zone.

Uncorrected near (UNVA) and distance (UDVA) visual acuity, and corrected near (CNVA) and distance (CDVA) visual acuity, were assessed using Snellen visual charts for distance vision and the Jaeger Scale for near vision, and then converted into a logarithm of the minimum angle of resolution (logMAR) notation. In all examinations, the eyes were not dilated. The examinations were performed at baseline, then postoperatively at 1 week, 1 month, 3 months and 6 months. Bilateral LASIK multifocal aspheric corneal ablation treatment was performed at least 6 months after cataract surgery.

The procedures were completed using the Supracor PresbyLASIK algorithm with a Teneo 317 excimer laser (Technolas Perfect Vision GmbH, Munich, Germany). The dominant eye of the patient was planned as plano for distance, while the non-dominant eye was slightly aimed at myopia of −0.5 D. However, we simulated the desired postoperative outcome before surgery using a contact lens. In the 14 patients included in the study, the distance setting was considered comfortable for the dominant eye. The LASIK incision was performed using a femtosecond laser (Ziemer Ophthalmic Systems, LDV, Port, Switzerland) with a target flap thickness of 110 μm. The hinge was set superior in each case, with a flap diameter of 9.5 mm.

**Figure 1:**
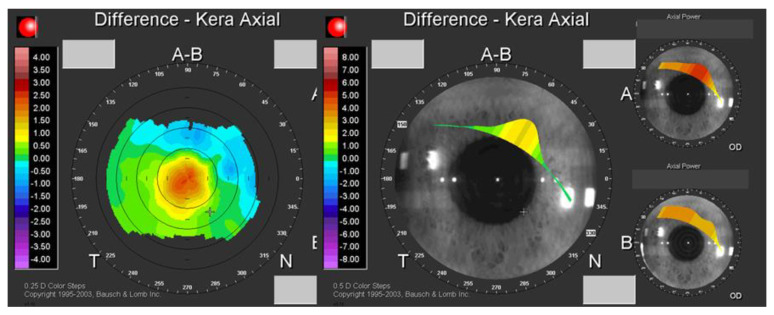
Topographical imaging showing a central steepness of the cornea created using the Supracor PresbyLASIK algorithm.

Our presbyLASIK protocol included a treatment algorithm with 2 phases. In the first phase, the dominant eye is treated for emmetropia and the non-dominant eye to aim for −0.5 diopters myopia with the excimer laser. The Munnerlyn formula [15] was used to determine the feasibility of the ablative process. In a second phase, but in the same treatment, a central steepness is achieved by ablation in a 3–6 mm zone and, in principle, is an addition for near vision. A multifocality is created in the cornea, which seamlessly represents a correction for near, intermediate and distance vision (Figure 1). Multifocality was created in both treated eyes during PresbyLASIK. To counteract the spherical aberration induced by this multifocal treatment, the laser applied additional wavefront-guided correction.

**Figure 2:**
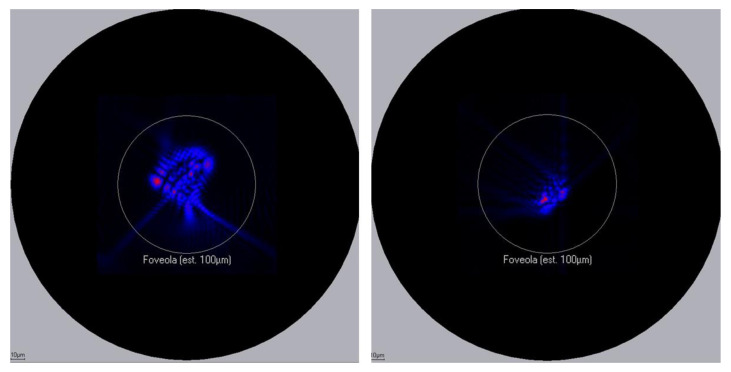
Reduction of PSF after wavefront-guided presbyLASIK.

This wavefront-guided correction reduces higher-order aberrations (HOA), as shown by the point spread function (PSF). It can be qualitatively appreciated in a patient example of how the PSF was significantly reduced after wavefront-guided presbyLASIK (Figure 2). The focus was mainly on the correction of spherical aberration.

The postoperative topical regime was Tobradex (Alcon Laboratories, Inc., Fort Worth, TX, USA) 3 times daily for seven days, and topical hyaluronic acid 0.15% 3 times daily for a month.

Statistical analysis was performed using the IBM SPSS Statistics version 22.0 (IBM Corp., Armonk, NY, USA). The Kolmogorov–Smirnov and Shapiro–Wilk tests were used to test the data sets for normal distribution. If *p* > 0.05, the data set was considered normally distributed. If the data sets were parametric, they were calculated using the Pearson normality test, an unpaired t-test and ANOVA test. For the non-parametric data sets, the Friedmann test was used for further analysis. Significance was considered to be when *p* < 0.05.

## 3. Results

A total of 28 eyes in 14 patients were treated, of which 9 (64%) were female and 5 (36%) were male. The mean age was 56 ± 13 years. Mean preoperative MRSE was −1.43 ± 1.03 D (range: −0.50 to −3.0 D), mean sphere −0.87 ± 0.81 D (range: −2.00 to 0.25 D) and mean cylinder −1.13 ± 0.73 D (range: 0.00 to −2.00 D). The mean preoperative monocular CDVA was 0.1 ± 0.85 logMAR (Snellen) and the CNVA, 0.19 ± 0.82 logMAR (Jaeger 5).

### 3.1. Dominant Eye

#### 3.1.1. Refraction

**Figure 3:**
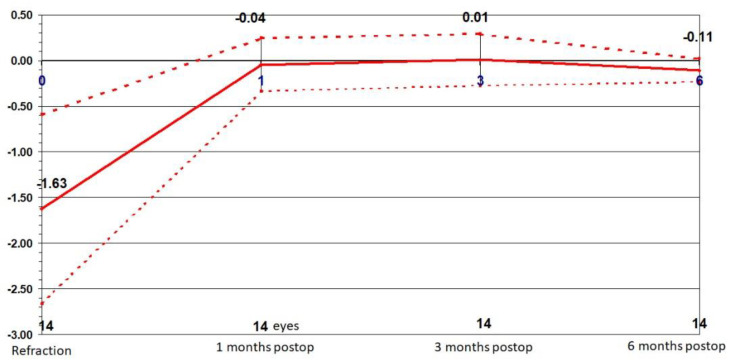
Stability: Change in refraction over time in the dominant eye.

The mean preoperative refraction in the dominant eye was −1.63 ± 1.03 D. Distance adjustment was aimed for emmetropia. The mean postoperative refraction was −0.04 ± 0.29 D, 0.01 ± 0.28 D and −0.11 ± 0.13 D after 1, 3 and 6 months, respectively (Figure 3). Refraction significantly improved from preoperative testing to 1-month postoperative testing (*p* = 0.001), without further significant changes at later times (*p* = 0.37) and (*p* = 0.42).

#### 3.1.2. Distance Vision

**Figure 4:**
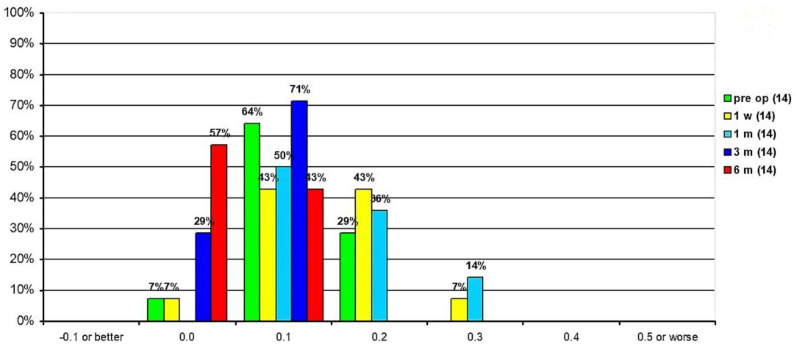
Uncorrected distance visual acuity percentage distribution.

At the 1-week postoperative test, 93% of eyes had a UDVA better than 0.2 logMAR, while the remainder had better than 0.3 logMAR. At 1 month after surgery, 50% of eyes had a UDVA of 0.1 logMAR, 36% had a UDVA of 0.2 logMAR, and the remainder (14%) had a UDVA of 0.3 logMAR. These same percentages persisted at 3 months, postoperatively (50%, UDVA of 0.1 logMAR; 36%, 0.2 logMAR; 14%, 0.3 logMAR. At 6 months, postoperatively, 57% of eyes had UDVA of 0.0 logMAR and the remainder of eyes (43%) had a 0.1 logMAR (Figure 4). Preoperative UDVA was 0.42 ± 1.15 logMAR and increased to 0.14 ± 1.05 logMAR, 0.21 ± 1.10 logMAR, 0.07 ± 1.05 and 0.05 ± 1.00 logMAR 1 week, 1 month, 3 and 6 months postoperatively, respectively. Compared to the preoperative value, the increase to each postoperative value was significant (*p* < 0.001). Visual acuity significantly increased between the first postoperative week and the third postoperative month (*p* = 0.014) and from the first postoperative month to the third postoperative month (*p* = 0.017).

The mean CDVA was 0.10 ± 0.89 logMAR preoperatively. CDVA fluctuated from 0.12 ± 0.83 logMAR at 1 week postoperatively, 0.14 ± 1.05 logMAR at 1 month postoperatively, 0.05 ± 1.0 logMAR at 3 months postoperatively, to 0.03 ± 1.05 logMAR at 6 months postoperatively. CDVA increased significantly from preoperative values to those obtained at 3 months (*p* = 0.027) and 6 months (*p* = 0.003), postoperatively. There was also a significant increase in CDVA from the first postoperative week to the third postoperative month (*p* = 0.003), and the sixth postoperative month (*p* < 0.001). CDVA also significantly increased from the first postoperative month to the third postoperative month (*p* < 0.001) and to the sixth postoperative month (*p* < 0.001). There was no significant change in CDVA after the third postoperative month.

**Figure 5:**
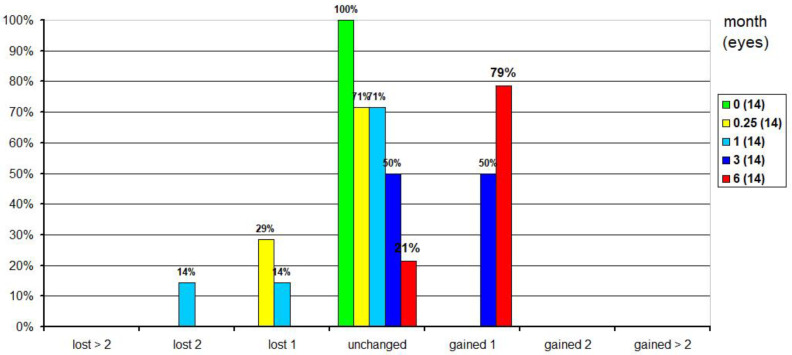
Safety: Changes in corrected distance visual acuity over time compared to preoperative values.

CDVA was also assessed in terms of safety. At 1 week postoperatively, CDVA was unchanged in 71% of eyes, while 29% lost 1 line of CDVA. At 1 month postoperatively, CDVA was unchanged in 71% of eyes, but 14% lost 1 or 2 lines of CDVA, respectively. At 3 months postoperatively, CDVA was unchanged in 50% of eyes, while the other half gained 1 line. By 6 months, 21% of eyes had unchanged CDVA and 79% gained 1 line (Figure 5).

**Figure 6:**
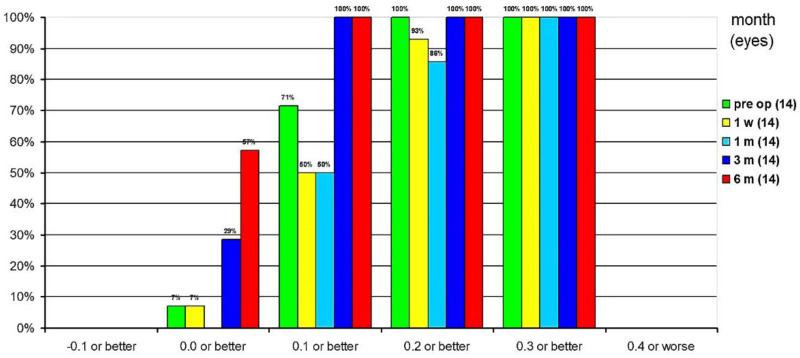
Cumulative corrected distance visual acuity.

The cumulative CDVA at 1 week postoperatively was 0.3 logMAR or better in all eyes, 0.2 logMAR or better in 93%, 0.1 logMAR or better in 50%, and 0 logMAR or better in 7% of eyes. At 1 month postoperatively, CDVA was 0.3 logMAR or better in all eyes, 0.2 logMAR or better in 86% and 0.1 logMAR or better in 50%. At 3 months postoperatively, CDVA was already 0.1 logMAR or better in all eyes, and 29% had CDVA of 0 logMAR or better. At 6 months postoperatively, all eyes had a 0.1 logMAR or better and 57% had a CDVA of 0 logMAR or better (Figure 6).

**Figure 7:**
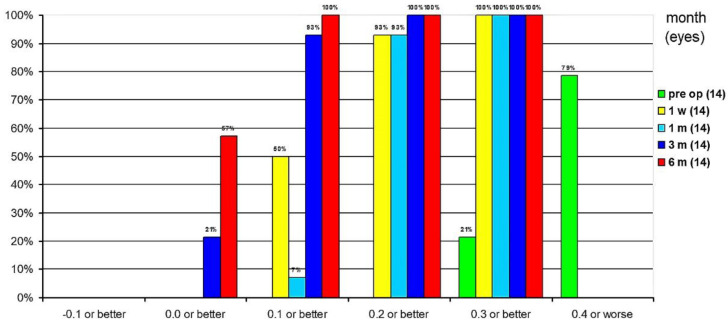
Cumulative uncorrected distance visual acuity.

The cumulative UDVA at 1 week postoperatively was 0.3 logMAR or better in all eyes, 0.2 logMAR or better in 93% of eyes, and 0.1 logMAR or better in 50% of eyes. One month postoperatively, UDVA was 0.3 logMAR or better in all eyes, 0.2 logMAR or better in 93% of eyes and 0.1 logMAR or better in 7% of eyes. Three months postoperatively, UDVA was already 0.2 logMAR or better in all eyes, 93% of eyes had UDVA of 0.1 logMAR or better and 21% of eyes had UDVA of 0 logMAR. At 6 months postoperatively, all eyes had a 0.1 logMAR or better and 57% of eyes had a UDVA of 0 logMAR or better (Figure 7).

#### 3.1.3. Near Vision

The mean preoperative UNVA was 0.46 ± 1.0 logMAR. At 1 week postoperatively UNVA increased to 0.13 ± 0.77 logMAR, 1 month postoperatively to 0.09 ± 0.89 logMAR, 3 months postoperatively to 0.14 ± 0.89 logMAR and 6 months postoperatively to 0.15 ± 0.89 logMAR. The difference in UNVA was significant at each time point compared to preoperative values (*p* < 0.001).

The CNVA was 0.19 ± 0.80 logMAR preoperative. One week postoperatively, there was a slight visual improvement to 0.10 ± 0.89 logMAR, 1 month postoperatively to 0.08 ± 1.04 logMAR, 3 months postoperatively 0.10 ± 1.0 logMAR and 6 months postoperatively to 0.10 ± 0.96 logMAR. Visual acuity only increased significantly between the preoperative values and 1 month postoperatively (*p* = 0.007). Otherwise, there was no other significant improvement compared to preoperative values (*p* > 0.05).

### 3.2. Non-Dominant Eye

#### 3.2.1. Refraction

**Figure 8:**
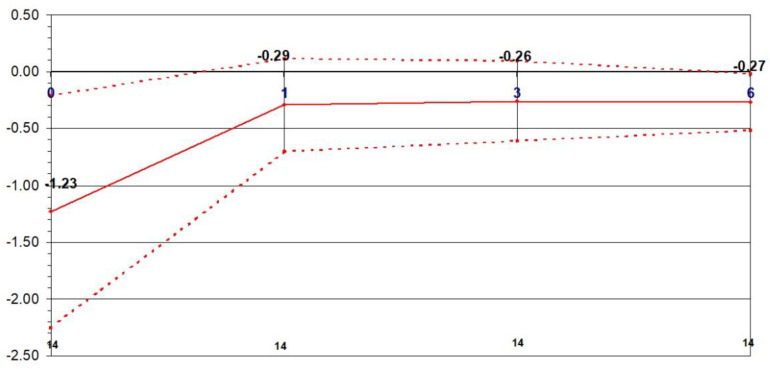
Stability: Change in refraction over time in the non-dominant eye.

The refraction target value was set at −0.5 D. The mean preoperative refraction was −1.23 ± 1.03 D, −0.29 ± 0.35 D at 1 month postoperatively, −0.26 ± 0.32 D at 3 months postoperatively and −0.28 ± 0.22 D at 6 months postoperatively. Refraction significantly changed at 1 month (*p* = 0.001), 3 months (*p* = 0.004) and 6 months (*p* = 0.002), compared to preoperative values (Figure 8).

#### 3.2.2. Distance Vision

**Figure 9:**
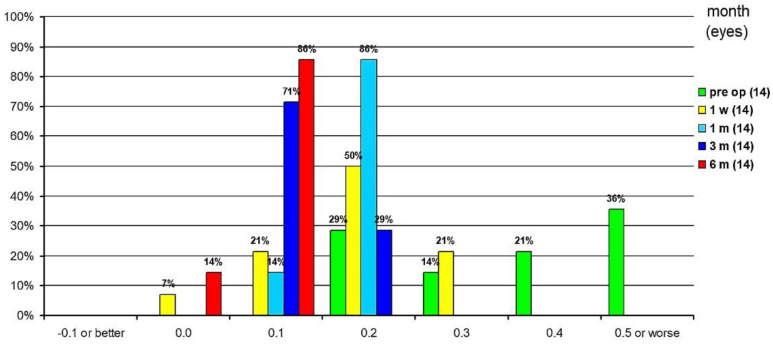
Distribution of uncorrected distance visual acuity.

At 1 week postoperatively, 7% of eyes had an uncorrected distance visual acuity (UDVA) of 0 logMAR, 21% of 0.1 logMAR, 50% of 0.2 logMAR and 21% of 0.3 logMAR, respectively. By 1 month after surgery, 14% of eyes had a UDVA of 0.1 logMAR and 86% had a UDVA of 0.2 logMAR. At 3 months postoperatively, 71% of eyes had a UDVA of 0.1 logMAR and 29% of 0.2 logMAR. Six months postoperatively, 14% had a UDVA of 0 logMAR and 86% of 0.1 logMAR (Figure 9). The mean UDVA was 0.33 ± 0.82 logMAR preoperatively, and increased to 0.17 ± 0.85 logMAR, 0.19 ± 1.30 logMAR, 0.12 ± 1.15 logMAR and 0.1 ± 1.40 logMAR at 1-week, 1-, 3- and 6-month postoperative examinations, respectively. Compared to preoperative values, there was a significant improvement in UDVA after 3 and 6 months postoperatively (*p* < 0.001). Between the first postoperative week and the third and sixth postoperative month, there was a significant improvement in UDVA (*p* = 0.031 and *p* = 0.004). Between the first and third postoperative months, there was an improvement in UDVA (*p* = 0.01). All other parameters were not significant.

The mean CDVA was 0.11 ± 0.85 logMAR preoperatively, 0.13 ± 0.77 logMAR, 0.12 ± 1.10 logMAR, 0.08 ± 1.05 logMAR and 0.05 ± 1.0 logMAR at 1-week, 1-, 3- and 6-month postoperative examinations, respectively. A significant increase in CDVA was observed between preoperative tests and 6 months postoperatively (*p* = 0.02). From the first postoperative week to the third postoperative month (*p* = 0.01), and to the sixth postoperative month (*p* < 0.001), there was a significant increase in CDVA. Compared to the first postoperative month, CDVA significantly increased at the third postoperative month (*p* = 0.036), and at the sixth postoperative month (*p* = 0.002). All other tested parameters to CDVA did not vary significantly with time.

**Figure 10:**
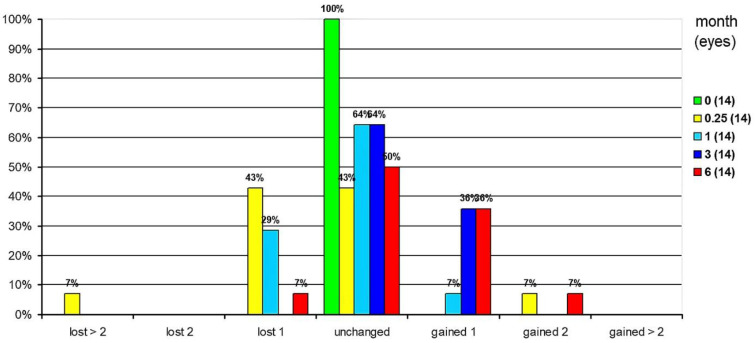
Safety: Changes of corrected distance visual acuity in percentage.

CDVA was assessed in terms of safety. At 1 week postoperatively, 43% of eyes were unchanged, 43% lost 1 line of CDVA, and 7% 2 lines. At 1 month postoperatively, 64% of eyes were unchanged. While 7% of eyes gained 1 line of CDVA, 29% lost 1 line of CDVA. At 3 months postoperatively, 64% of eyes were unchanged and 36% gained 1 line of CDVA. By 6 months, 50% of eyes were unchanged, 36% gained 1 line and 7% gained 2 lines of CDVA (Figure 10).

**Figure 11:**
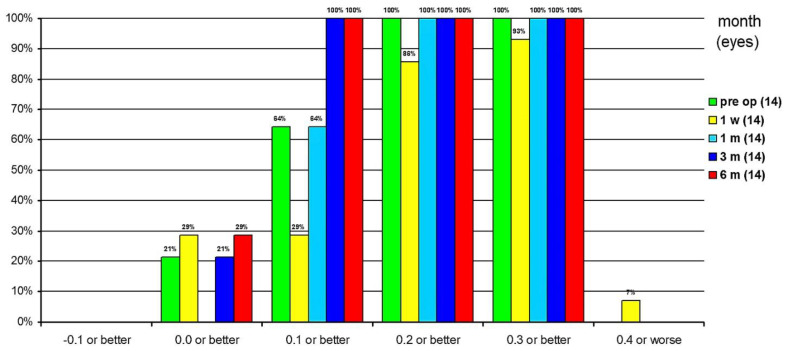
Cumulative corrected distance visual acuity in percentage.

The cumulative CDVA at 1 week postoperatively was 0.4 logMAR or worse in 7% of eyes, 0.3 logMAR or better in 93%, 0.2 logMAR or better in 86%, 0.1 logMAR or better in 29% and 0 logMAR or better in 29%. At 1 month postoperatively, CDVA was 0.2 logMAR or better in all eyes and 0.1 logMAR or better in 64%. At 3 months postoperatively, CDVA was already 0.1 logMAR or better in all eyes, and 21% had CDVA of 0 logMAR or better. At 6 months postoperatively, all eyes had a 0.1 logMAR or better and 29% had a CDVA of 0 logMAR or better (Figure 11).

**Figure 12:**
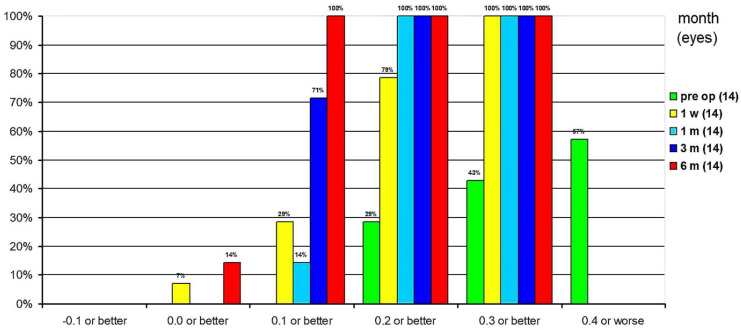
Cumulative uncorrected distance visual acuity.

The cumulative UDVA at 1 week postoperatively was 0.3 logMAR or better in all eyes, 0.2 logMAR or better in 79% of eyes, 0.1 logMAR or better in 29%, and 0 logMAR or better in 7%. One month postoperatively, UDVA was 0.2 logMAR or better in all eyes, and 0.1 logMAR or better in 14% of eyes. Three months postoperatively, UDVA was already 0.2 logMAR or better in all eyes, and 71% of eyes had a UDVA of 0.1 logMAR or better. At 6 months postoperatively, all eyes had a 0.1 logMAR or better and 14% had a UDVA of 0 logMAR or better (Figure 12).

#### 3.2.3. Near Vision

The mean UNVA was 0.54 ± 1.22 logMAR preoperatively. Postoperative examinations at 1 week, then at 1, 3, and 6 months, revealed that near vision increased to 0.10 ± 0.82 logMAR, 0.09 ± 0.89 logMAR, 0.1 ± 0.77 logMAR and 0.11 ± 0.80 logMAR, respectively. Compared to the preoperative values, postoperative visual acuity increased significantly at each time point (*p* < 0.001).

The mean preoperative CNVA was 0.19 ± 0.82 logMAR. It increased to 0.08 ± 1.05 logMAR 1 week post-operatively and remained stable at each time point until at least 6 months, the last time they were tested. Postoperative CNVA values were significantly better than preoperative values (*p* = 0.02).

### 3.3. Higher-Order Aberrations

The RMS of higher-order aberrations (RMS-HOA) (at 6 mm diameter) increases in mean by 0.07 ± 0.1 µm from preoperatively to 6 months postoperative values (*p* = 0.04), which is significant. Customized treatment decreased the spherical aberration (Z400) decrease in mean by 0.36 ± 0.12 µm (*p* < 0.001). Quatrefoil aberrations (Z440) decreased significantly at 6 months (*p* < 0.001), compared to the preoperative values in mean, by 0.29 ± 0.11 µm (*p* < 0.001). The total coma RMS did not significantly increase in mean, changing by 0.03 ± 0.07 µm (*p* = 0.35). The total trefoil RMS did not significantly decrease, changing by 0.02 ± 0.05 µm (*p* = 0.28).

## 4. Discussion

In our study, postoperative UDVA and UNVA were better than preoperative values for all our patients. All patients in the study could be considered as spectacles-free for driving or for reading in standard conditions.

Most ophthalmologists consider that having a residual accommodation is an advantage for near visual acuity [16], and for some, it might be considered a necessary capacity for good outcomes. Herein, it could be demonstrated that even without any residual accommodation—all of our patients were pseudophakic—it is possible to obtain a monocular UNVA of almost 0.1 logMAR and 0.15 logMAR with PresbyLASIK in non-dominant and dominant eyes, respectively. Indeed, the target refraction values of the dominant eye (set to plano) were achieved, as confirmed by the visual acuity of 0.1 logMAR or more at 6 months postoperatively. As expected, UNVA was slightly lower, averaging 0.14 logMAR at 6 months after surgery. In the non-dominant eye (set to −0.5 D), the target refraction value of −0.5 D was not perfectly reached; we found a slight overcorrection with a mean postoperative refraction value of −0.27 D at 6 months postoperatively. This led to a rather high uncorrected distance vision of at least 0.1 logMAR. The uncorrected near vision was 0.11 logMAR due to the slight overcorrection, which was only slightly better than in the dominant eye. Satisfaction was high among all patients, because the patients were very well informed and knew what to expect. However, it must be taken into account that the situation could be somewhat different with a larger group of patients. In any case, patient information is eminently important, especially for PresbyLASIK.

Conventional femto-PresbyLASIK is always a compromise between distance and near correction, as it creates unwanted aberrations in the pupillary region, especially spherical aberrations. If not taken into account, spherical aberrations might lead to an unsatisfactory quality of vision. With the wavefront-guided treatment, however, spherical aberrations could decrease, giving improved visual outcomes. In our study, we attempted to account for the most clinically relevant aberrations, namely, spherical aberrations (Z400). The approach allowed us to significantly decrease Z400 aberrations, even in a multifocal treatment, which usually frequently induces spherical aberrations. The Z300 (i.e., coma and trefoil) aberrations are not usually affected during multifocal treatment, as long as it is centered on the corneal apex. Therefore, we excluded patients with a kappa angle over 6°. Wavefront-guided treatments are also apparently effective in correcting the aberration induced not only by the cornea but also by the intraocular lens [17].

Similar studies have been made recently to determine the feasibility of presbyopia correction using LASIK technologies. A recent study used an aspheric ablation profile to increase spherical aberrations and enhance near vision associated with a micro-monovision [18]. However, as expected, increasing spherical aberration was associated with a decrease in far vision (UDVA of 0.08 logMAR at 6 months), with a micro-monovision that was not well tolerated in 4% of patients.

If we compare our pseudophakic population to phakic patients having undergone a wavefront-guided presbyLASIK treatment, we obtain quite similar results in terms of UDVA and UNVA. In another study, a UDVA and a UNVA of 0.22 logMAR and 0.30 logMAR, respectively, and a 0.1 logMAR Snellen equivalent in the non-dominant eye, were achieved [19].

Compared to the treatment of presbyopia with multifocal IOLs, which is considered to be already established, wavefront-guided PresbyLASIK is a newer surgical technique, with the advantage that it is less invasive than implanting an IOL, because the treatment is applied on the eye surface. On the other hand, the application of wavefront-guided PresbyLASIK is much more demanding regarding the indication and interpretation of the objective preoperative topographic and wavefront analyses, which have a high influence on the surgical result. Wavefront-guided femto-PresbyLASIK significantly alters the biomechanical and optical properties of the cornea, which have a major influence on the surgical outcome. It is not sufficient to apply the general LASIK criteria for the indication of wavefront-guided femto-PresbyLASIK. This is probably also the reason for the not universally encouraging treatment results, because the surgical indication was made on the basis of the LASIK criteria, and the corresponding refraction and other parameters from the wavefront analysis were not taken into account. In this sense, it can be assumed that not only the suitable patients received wavefront-guided femto-PresbyLASIK treatment [11].

Our study has a few limitations. First, there is a need for an adequately sized randomized trial, since our study was a retrospective analysis with a limited number of participants. However, it must be emphasized that all patients included in this study were in a pseudophakic state, and only later requested enhancement after cataract surgery had been performed. Indeed, an alternative would have been to perform a LASIK treatment with a so-called monovision LASIK (i.e., one eye for distance and the other for near vision). In this technique, the patient selection is less crucial [20], but the non-dominant eye has a drastic decrease in far vision, and stereoscopic vision is impacted [21]. Therefore, up to 15% of patients who undergo monovision LASIK may be dissatisfied [22]. Second, we could not compare our patient population to patients who had multifocal lens implantation. Therefore, extrapolating our results to this population of patients is not possible.

The industry and researchers should focus on more accurate or innovative wavefront-guided PresbyLASIK protocols, especially addressing the needs of pseudophakic patients, as this problem affects a large number of people in their early sixties, and could substantially improve the quality of life for these patients. Even if our results are very encouraging, there is still some room for progress. It is conceivable that an entire eye-adapted treatment protocol could be developed for each patient, based on the spherical aberration of the cornea and the IOL.

Clinicians should be aware of more precise refractive outcomes after LASIK in patients with a monofocal IOL than a multifocal IOL [23]. If the patient is not carefully selected, the surgical outcome may be worse with a multifocal IOL due to the larger optical aberrations and reduced targeting accuracy compared to LASIK. Indeed, using a laser to adapt the size of the optic and transition zone might offer a more customized treatment profile [24].

Finally, clinicians should also manage patient expectations and anxiety. In our study, UDVA fluctuated a lot during the first six months. This is a natural phenomenon caused by corneal remodeling. Initially, the multifocal treatment plan leads to light myopization, and UDVA decreases. Then the corneal epithelium compensates for the irregular corneal shape induced by laser treatment by flattening the surface, which is associated with an emmetropic shift and a slight decrease in near visual acuity. Near-vision decrease was very low, at 6 months in the dominant eye, and insignificant in the non-dominant eye.

## 5. Conclusions

Steepening the central cornea with wavefront-guided PresbyLASIK creates a corneal multifocality, which improved near vision in both eyes. The procedure was safe, as postoperative spherical aberration was within acceptable limits. Wavefront-guided femto-PresbyLASIK offers the possibility of treating presbyopia in pseudophakic eyes without having to perform intraocular surgery.

## Figures and Tables

**Table 1:** Details regarding preoperative data prior to PresbyLASIK, inclusive preoperative refraction, visual acuity in logMAR, and time elapsed since cataract surgery in months.

	Mean + SD	Range
Age	55.9 ± 13.1 Years	(Range 37–74)
UDVA before: Dominant eye	0.42 ± 1.13	0.3–0.5
CDVA before: Dominant eye	0.10 ± 0.87	0.0–0.2
UNVA before: Dominant eye	0.45 ± 1.0	0.7–0.3
CNVA before: Dominant eye	0.19 ± 0.81	0.3–0.1
UDVA before: Non-dominant eye	0.33 ± 0.83	0.5–0.2
CDVA before: Non-dominant eye	0.11 ± 0.85	0.2–0.0
UNVA before: Non-dominant eye	0.53 ± 1.2	0.7–0.4
CNVA before: Non-dominant eye	0.20 ± 0.83	0.3–0.1
Preoperative refraction: dominant eye	−1.14 ± 0.73 sph −0.96 ± 0.77 cyl −1.62 ± 1.03 sph aquiv	−2.0–−0.25 −2.0–0 −3.0–−0.5
Preoperative refraction: non-dominant eye	−0.59 ± 0.81 sph −1.23 ± 0.67 cyl −1.23 ± 1.03 sph aquiv	−1.75–±0.25 −2.0–−0.25 −2.63–0.13
Time elapsed since the cataract surgery (months)	8.93 ± 3.82	6–16

## Data Availability

The data presented in this study are available on request from the authors; the datasets, in particular, are archived in the clinics treated. The data are not publicly available as they contain information that could compromise the privacy of the participants.
